# Metabolic flare phenomenon mimicking disease progression on ^18^Flouride– Fluorodeoxyglucose PET/CT scan in breast cancer treated with paclitaxel-based chemotherapy

**DOI:** 10.22038/AOJNMB.2022.68237.1474

**Published:** 2023

**Authors:** Vijay Singh, Shivangi Dikshit, Manish Ora, Aftab Hasan Nazar

**Affiliations:** Department of nuclear medicine SGPGIMS, India

**Keywords:** Flare phenomenon, Paclitaxel, Breast cancer

## Abstract

^18^F-fluorodeoxyglucose Positron emission tomography (^18^F-FDG PET/CT ) is now being used as a single modality for metastatic workup and response evaluation in breast cancer. An increase in metabolic activity indicates disease progression; however, metabolic flare should be kept in mind. Metabolic flare is a well-documented phenomenon reported in metastatic breast and prostate cancer. Despite a favorable response to therapy, there is a paradoxical increase in radiopharmaceutical uptake. The flare phenomenon with various chemotherapeutic and hormonal agents is well acknowledged in bone scintigraphy. However, very few cases have been documented on PET/CT. Increased uptake may be noted after treatment is instituted. The increased osteoblastic activity is associated with the healing response of bone tumors. We report a case of treated breast cancer. She presented with metastatic recurrence after four years of initial management. The patient was started on paclitaxel chemotherapy. Serial ^18^F- FDG PET/CT demonstrated metabolic flare and complete metabolic response.

## Introduction

 Breast cancer has an incidence rate of over 2.2 million cases per year. Worldwide it is the most common cancer among females (1). There has been an advancement in the screening and management of breast cancer in the last few decades. Unlike many other lethal cancers, it is now considered manageable if diagnosed early in the course of the disease. However, metastatic disease is highly fatal, as it causes more than 0.6 million deaths yearly (1). These metastasis lesions involve vital organs and become clinically apparent. It remains a challenge to treat them. Ultimately results in resistance to the currently available systematic therapies. Consequently, managing systemic metastasis is essential in the management of breast cancer.

 The possibility of breast cancer developing is increased by several risk factors, such as sex, aging, estrogen, family history, gene mutations, and poor lifestyle. (2). mammography is widely used to screen for breast cancer. It leads to early detection and reduced mortality. Magnetic resonance imaging (MRI) is more sensitive than mammography as a screening method. It has been implemented and studied during the last decade (3). Diagnostic workup includes mammography, ultrasonography, and biopsy. 

 Approximately one-third of the patients present with metastasis involving any organ. Breast cancer frequently metastasizes to the bone, lung, liver, and brain (4). Metastatic breast cancer (MBC) affects bone in 30-60% of patients, followed by the liver (15-32%). The lung (21-32%) and brain (4-10 %) are other commonly involved organs (4). Computed Tomography (CT) and FDG PET/CT are performed in suspicious metastasis.

 Breast cancer is an example of multi-modality cancer management, including surgery, chemotherapy, radiation therapy, hormonal therapy, and newer biological therapies. Systemic therapies facilitate the management of MBC (5). The endocrine treatment reduces estrogen production and blocks signalling through the estrogenic receptor or antagonizes the receptor.

 FDG PET/CT is increasingly used to detect distant metastasis and to monitor treatment response. It measures tumor glycolysis, an indirect measure of cell proliferation (6). A paradoxical increase in FDG PET/CT uptake in bone metastases metabolic activity after endocrine treatment has been proposed as a marker of therapy efficacy(7). However, it remains difficult to differentiate from the disease progression. It represents a pitfall in the image interpretation (8).

***Case Presentation***

**Figure 1:**
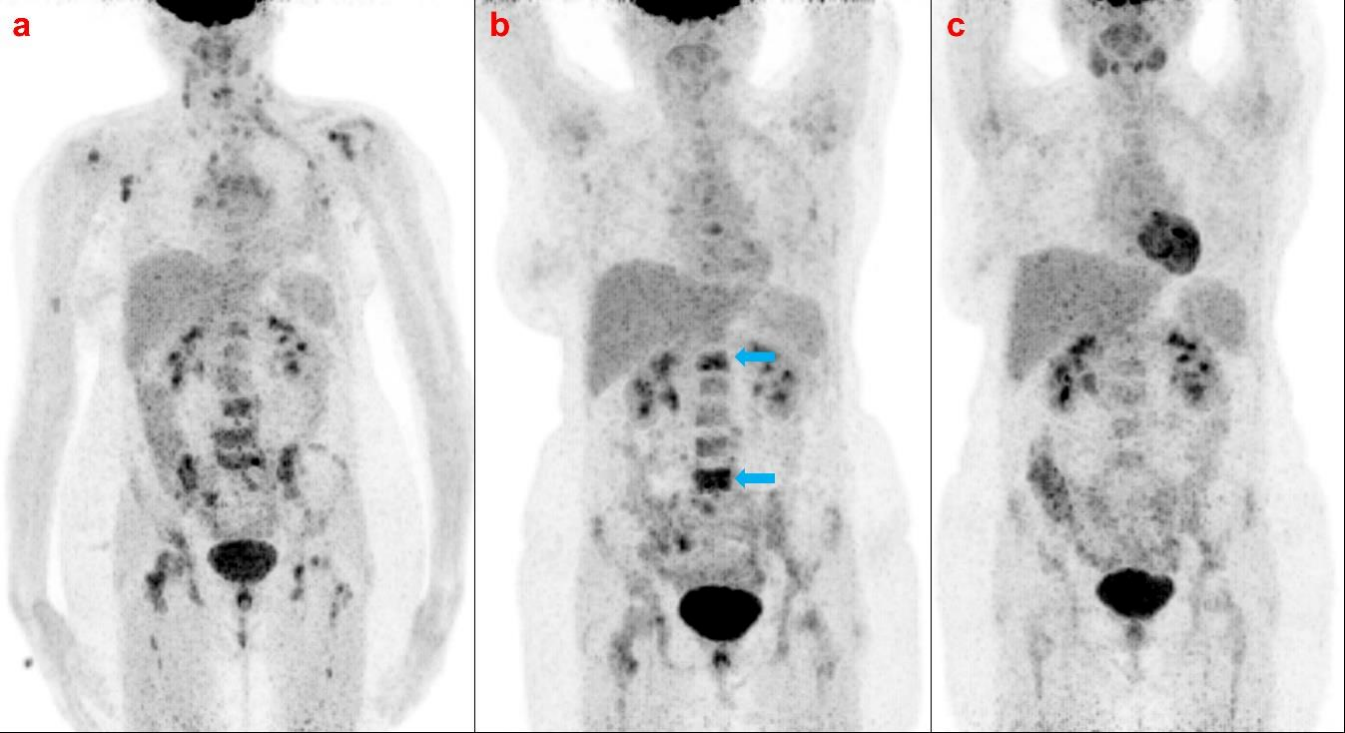
Maximum Projection Image (MIP) of the patient on Baseline PET/CT (**a**) reveal tracer uptake in multiple vertebrae, Interim PET/CT MIP image (**b**) reveals increased tracer uptake in vertebrae, MIP image on End Cycle PET/CT (**c**) reveal negligible uptake in the corresponding vertebrae

**Figure 2:**
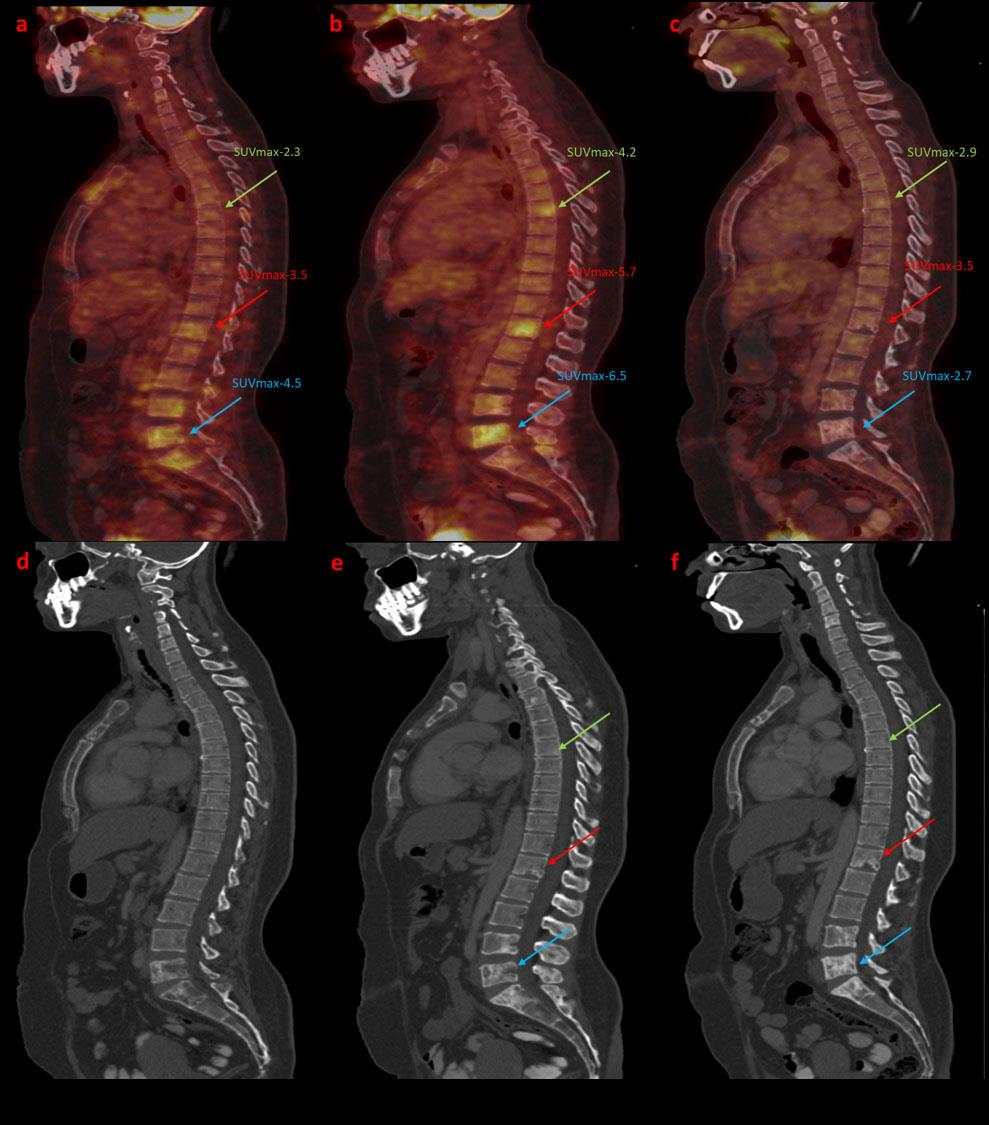
The initial FDG PET scan (Figure 1a-maximum intensity projection [MIP], **2a**- sagittal fused PET-CT, **2d**- sagittal CT) showed metabolically active mediastinal, right axillary, right supraclavicular, cervical, and retroperitoneal lymph nodes with extensive skeletal lesions. Interim FDG PET scan (Figure 1b-MIP, **2b**-sagittal fused PET-CT) showed an increase in the FDG uptake and the SUV_max_ in the D7 (**Green arrow**), L1 (**Red arrow**), and L5 (**Blue arrow**) vertebrae. The SUV_max_ for vertebral lesions was 4.2, 5.7, and 6.5, respectively. Above mentioned corresponding vertebral lesions shows lytic -sclerotic changes on the CT (Figure **2e**, Sagittal CT). The patient underwent an end of cycle FDG PET scan (Figure 1c-MIP, **2c**-sagittal fused PET-CT) after four more cycles of chemotherapy. There was no significant FDG avidity in the previously noted lesion. Dense sclerotic changes were noted on CT (Figure **2f**-sagittal CT)

 A 47-year-old female presented with a left breast mass five years back; histopathology suggested breast carcinoma. She received eight-cycle of neo-adjuvant chemotherapy (NAC) (4-cycle Cyclophosphamide, Epirubicin, and 5-Fluorouracil followed by four-cycle Docetaxel). After neoadjuvant chemotherapy, a left radical mastectomy was done, followed by chest wall radiotherapy. She presented with recent onset back pain. X-rays of the lumbosacral region were normal. She was referred for a whole-body FDG PET-CT to rule out metastasis. The whole body FDG PET scan (Figure 1-2) showed metabolically active regional and other non-regional lymph nodes and multiple skeletal metastases in Dorso-lumbar vertebrae. She underwent three cycles of paclitaxel-based chemotherapy.

 The whole body interim FDG PET scan shows significant resolution of the lymph nodes. However, the skeletal lesions show increased FDG uptake (Figure 1-2). There was no significant difference in injected activity or image acquisition time between the two scans. Sclerotic changes appeared on the CT. A possible metabolic flare diagnosis was proposed based on CT appearance and partial therapy response in the lymph nodes. The patient continued on the same chemotherapy regime. She received four cycles of chemotherapy and underwent an end-of-treatment FDG PET/CT scan for response evaluation (Figure 1-2). It revealed a complete metabolic response. Dense sclerotic changes were noted on CT with no metabolic uptake, indicating a healing response. The patient has been on follow-up for one year. She has no clinical evidence of active disease. 

## Discussion

 Among female cancers, breast cancer has the highest incidence rate (1). The prognosis of breast cancer patients is generally favorable due to early detection and comprehensive treatment. However, 20–30% of patients eventually develop MBC. These patients have a poor prognosis with a median survival of two years (4). Breast cancer commonly metastasizes to bone, liver, lung, and brain (4). Breast cancer is widely acknowledged as a heterogeneous disease in terms of the metastatic capacity of the primary tumor and time to disease metastasis. Tumor size, histologic grade, nodal stage, and receptor status influence metastasis (4). Perou et al. (9). described breast cancer molecular subtypes based on a specific gene expression pattern and divided them into four simple subtypes based on hormone receptor (HR) and human epidermal growth factor receptor 2 (HER2) status: HR+/HER2-, HR+/HER2+, HR-/HER2+, and triple-negative (TN). These subtypes have differences in prognosis and adjuvant therapy response (4).

 Breast cancer, unlike many other lethal tumors, is now considered a manageable disease if diagnosed early in its progression. However, metastatic illness is incurable, causing over 0.6 million deaths annually (1). Previous research has shown that early breast cancer detection coupled with appropriate treatment could significantly reduce breast cancer mortality rates over the long run. Mammography is the current gold standard for breast cancer screening. However, it is less effective for women under 40 and those with dense breasts, less sensitive to small tumors (less than 1 mm, around 100,000 cells), and does not indicate disease progression(3). Contrast-enhanced digital mammography provides greater diagnostic accuracy than mammography and ultrasonography in dense breasts. However, it is not commonly available due to its high cost and radiation exposure (3). Ultrasound has been utilized as a supplement to mammography as a medical imaging tool (3). MRI can detect small lesions that mammography cannot identify; nevertheless, it is expensive and has low specificity, leading to over diagnosis. Recent years have seen the development of microwave imaging (MI) methods that could replace mammography as a less invasive and more cost-effective method of diagnosing breast cancer. However, it is still in the research phase (3). FDG PET/CT is the most precise tool for visualizing the spread of malignancies or their response to treatment (6). In the appropriate patient population, FDG PET/CT scanning has efficacy superior to that of conventional imaging for detecting locoregional and metastatic spread. For patients needing whole-body stagings, such as those with diseases in clinical stage 2b or higher, FDG PET/CT has been proposed as a one-stop imaging approach. It provides prognostic information and monitors response to therapy (6).

 The primary goals of treatment for nonmetastatic breast cancer are eradicating tumors and nearby lymph nodes and preventing recurrence. Local therapy consists of surgical excision and lymph node sampling or removal, with the possibility of postoperative radiation therapy. Systemic treatment (Taxanes, Aromatase inhibitor, Paclitaxel, Docetaxel, Adriamycin, Cyclophosphamide, Carboplatin) may be administered either preoperatively (NAC) or postoperatively (adjuvant) or both (5). 

 The subtype of breast cancer dictates the standard systemic therapy administered, which consists of endocrine treatment for all HR+ tumors (with some patients also requiring chemotherapy), trastuzumab-based ERBB2-directed antibody therapy plus chemotherapy for all ERBB+ tumors (with the endocrine treatment given in addition, if concurrent HR positivity), and chemotherapy alone for triple-negative breast cancer(5). Currently, MBC remains incurable for most patients. The therapeutic objectives are life extension and symptom management. MBC utilizes the same broad categories of systemic therapy as neoadjuvant/adjuvant methods. Local therapeutic techniques (surgery and radiation) are often employed for palliation (5).

 Current treatment response criteria for MBC are based on tumor size measurements, often obtained using CT (10). However, metabolic markers determined by FDG PET/CT may be a more accurate predictor of therapy response than anatomical changes (11). Studies evaluating treatment responses in MBC are less common than those considering responses to NAC. This may be because the clinicopathological examination is nearly always available after NAC to serve as a reference standard, whereas it is infrequently available after treatment for MBC. Initial studies revealed that FDG PET/CT could distinguish between response and nonresponse to therapy after just one to three cycles (12). Recent studies also demonstrated that FDG PET is superior to CT for detecting response in osseous metastases, as FDG PET/CT is capable of detecting bony metastases before CT (13). 

 Sclerotic lesions presenting on CT following therapy may represent skeletal repair as opposed to new metastases, hindering an appropriate assessment of therapeutic response (13).

 Platinum-based chemotherapy has reported high tumor response in MBC; however, a few studies have reported metabolic flare (7, 8). 

 However, Flare reactions with paclitaxel chemotherapy are infrequently described (14). Tumor flare reaction, also known as "flare response," is an unexpected, transitory worsening of tumor-related symptoms after treatment. It does not imply that treatment is ineffective or disease progression. Flare response has been described as a clinical and metabolic flare. If patients have increased pain or a rise in the skin and soft tissue illnesses after starting therapy, the former is considered (8). While in the metabolic flare, an increased SUV is noted (8). Hormonal therapy and various conventional or second-line chemotherapy drugs have shown metabolic flare in breast cancer (7, 8, 15, 16). The apparent transient disease progression associated with the hormone-induced flare reaction results from an initial stimulation of tumor growth that precedes tumor regression. It is caused by temporary estrogen-like agonist effects triggered by increased hormone levels (17). 

 Tamoxifen has a partial estrogen-like stimulatory activity. After initiation of tamoxifen, a metabolic flare phenomenon may be noted in a few patients. It characterizes as transient increased FDG-PET tumor uptake after 7- 10 days of initiation of tamoxifen. This partial estrogen-like stimulatory activity of this antiestrogen may be especially apparent during the initial days of treatment when its levels are still low (18). The hormone-induced flare reaction may be the most reliable predictor of response. 75% to 90% of patients who experience a flare reaction show an objective response when their hormone treatment is continued (19). However, clinical flare reaction is only helpful to a limited extent as a predictor of hormone responsiveness. However, it may be impracticable to differentiate between a flare and the disease progression as it is recognized in less than 5% of patients. A flare reaction may occur in many more patients, but these patients do not exhibit any symptoms (20, 21). Hormone flare reactions are better predictors of hormone responsiveness than receptor assays. As flare demonstrates, the receptors are both there and working. The flare reaction is commoner in postmenopausal metastatic ER-positive breast cancer. Patients experience increased pain at metastatic sites within 7 to 10 days of beginning hormonal therapy. It may be accompanied by increased serum calcium, alkaline phosphatase, or tumour markers (18, 20, 21). Flare reaction with Taxol-based chemotherapeutic agent is seldomly reported (14). Taxol is an anti-microtubule agen. It causes mitotic arrest by binding to tubulin, the protein component of microtubules, and arrest mitotic growth, however, its mechanism in causing flare is not well understood. In a Phase II trial, Taxol was administered in advanced stage IV breast cancer. Seven of the 21 patients showed improvement in bone scan findings by 6-12 months after therapy, characterized by a decrease in the number or intensity of baseline lesions. Three of these seven had a flare response seen on the first scan after two cycles of Taxol (4-6 weak). It was characterized by increased activity in lesions and the appearance of new lesions, followed by subsequent improvement on follow-up scans (14). 

 However, flare reactions with Paclitaxel on FDG PET/CT have not been reported. Therefore FDG PET/CT performed within the first few months after Taxol chemotherapy that shows "worsening" need to be interpreted cautiously. Comparison with clinical and imaging modalities should be made to avoid misinterpretation of disease progression, as it may lead to treatment discontinuation or escalation.

 In this patient clinical improvement, metabolic resolution of the lymph nodes, and sclerosis of vertebrae with paradoxical increased FDG uptake suggest a possible flare reaction. Failure to correctly interpret this radiological feature could result in an incorrect description of progressive disease. Our case underlines the critical role of FDG PET/CT during therapy response and accurate interpretation of CT in hybrid PET/CT.
